# Large language model enhanced corpus of CO_2_ reduction electrocatalysts and synthesis procedures

**DOI:** 10.1038/s41597-024-03180-9

**Published:** 2024-04-06

**Authors:** Xueqing Chen, Yang Gao, Ludi Wang, Wenjuan Cui, Jiamin Huang, Yi Du, Bin Wang

**Affiliations:** 1 Laboratory of Big Data Knowledge, Computer Network Information Center, Chinese Academy of Sciences, Beijing, 100083 China; 2 University of Chinese Academy of Sciences, Beijing, 100049 China; 3 CAS Key Laboratory of Nanosystem and Hierarchical Fabrication, National Center for Nanoscience and Technology (NCNST), Beijing, 100190 China; 4 Hangzhou Institute for Advanced Study, UCAS, Hangzhou, 310000 China

**Keywords:** Electrocatalysis, Photocatalysis

## Abstract

CO_2_ electroreduction has garnered significant attention from both the academic and industrial communities. Extracting crucial information related to catalysts from domain literature can help scientists find new and effective electrocatalysts. Herein, we used various advanced machine learning, natural language processing techniques and large language models (LLMs) approaches to extract relevant information about the CO_2_ electrocatalytic reduction process from scientific literature. By applying the extraction pipeline, we present an open-source corpus for electrocatalytic CO_2_ reduction. The database contains two types of corpus: (1) the benchmark corpus, which is a collection of 6,985 records extracted from 1,081 publications by catalysis postgraduates; and (2) the extended corpus, which consists of content extracted from 5,941 documents using traditional NLP techniques and LLMs techniques. The Extended Corpus I and II contain 77,016 and 30,283 records, respectively. Furthermore, several domain literature fine-tuned LLMs were developed. Overall, this work will contribute to the exploration of new and effective electrocatalysts by leveraging information from domain literature using cutting-edge computer techniques.

## Background & Summary

CO_2_ electroreduction has garnered significant attention from both the academic and industrial communities, owing to its potential to effectively mitigate greenhouse gas emissions while simultaneously producing fuels and chemicals^1–3^. Its widespread adoption relies heavily on the development of efficient and reliable electrocatalysts. Over the past three decades, scientists have invested substantial efforts in the development of CO_2_ reduction electrocatalysts^4,5^; However, this trial-and-error approach has proven to be time-consuming and labor-intensive. Consequently, it becomes pivotal in accelerating catalyst development to establish a comprehensive database for CO_2_ electroreduction, which should encompass various information pertaining to the composition, synthesis, regulation, and performance of catalysts. Given the substantial workload involved, the manual annotation method by domain experts is deemed unreasonable. In recent years, emerging artificial intelligence (AI) technologies have exhibited tremendous potential in facilitating the construction of realm-specific datasets^6,7^. Extracting crucial information related to catalysts from domain literature is the initial step toward accelerating catalyst development using AI technologies. Traditionally, Named Entity Recognition (NER) methods have been employed for text mining and information retrieval^8–11^. However, NER often necessitates the establishment of algorithms tailored to specific tasks, which are typically undertaken by scientists or engineers with expertise in coding, data structures, and computer algorithms. Therefore, this approach is labor-intensive. Furthermore, NER algorithms are closely tied to their assigned tasks, lacking generalizable ability and thus making direct transfer to other tasks challenging. Additionally, extracted information tends to be intricate, heterogeneous, and diverse in the field of catalysis, leading to unsatisfied NER performance and reduced accuracy^12^. Therefore, the development and utilization of more general and robust methods for extracting domain knowledge are becoming increasingly imperative.

Recently, the emergence of large language models (LLMs), especially the widely acclaimed ChatGPT, has brought new prospects to the field of NER tasks^13^. It can be effectively operated by domain scientists who may not be well-versed in computer algorithms. However, ChatGPT is susceptible to information hallucinations, a glaring issue that significantly undermines its reliability in scientific domains^14–16^. Prompt engineering has proven to be a potential solution to mitigate the problem of artificial hallucinations^17–19^. For instance, Zheng *et al*. employed prompt engineering to guide ChatGPT in automating text mining for the synthesis conditions of metal-organic frameworks^17^. Nevertheless, the utility of this approach for more diverse and complex tasks within the catalytic science domain remains an area warranting further exploration. Moreover, the high demand for computing resources in LLMs also limits their application in various fields. The training and application of LLMs usually require a tremendous amount of computational power, which are not only expensive to purchase but also consume substantial amounts of electricity.

**Fig. 1:**
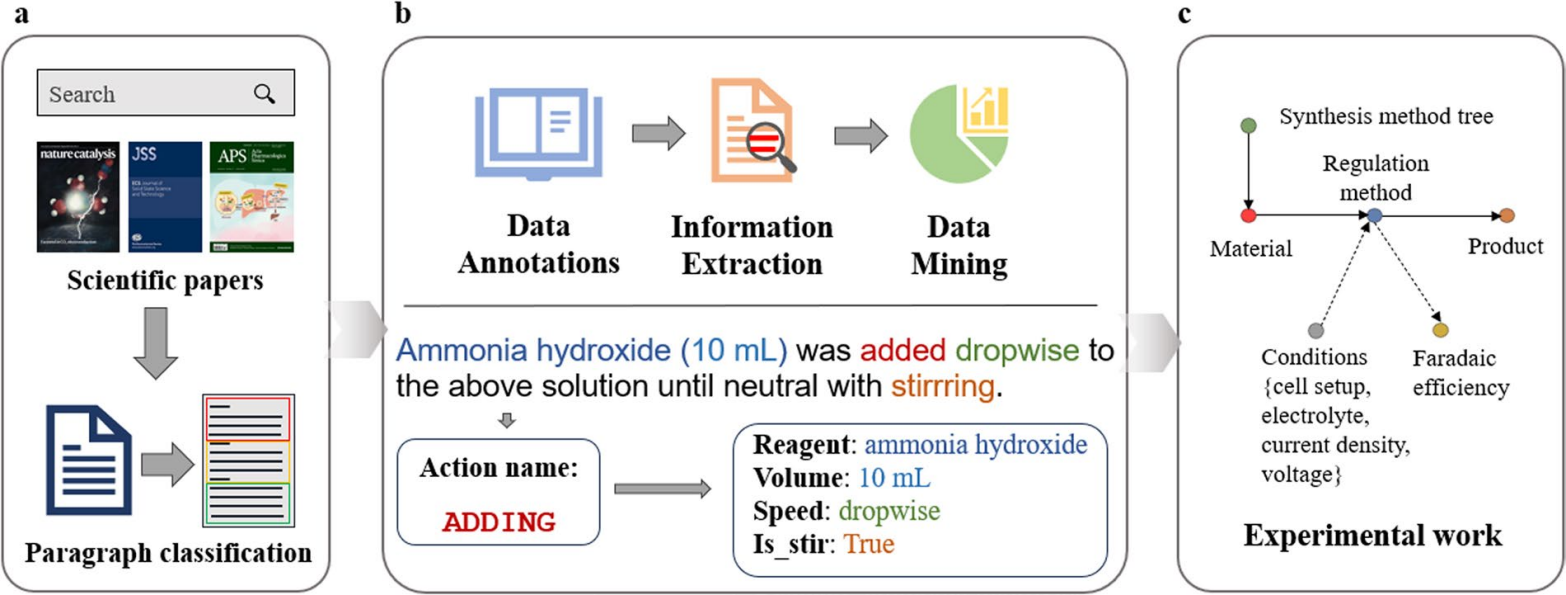
The schematic overview of dataset construction pipeline. (**a**) The process of literature search filtering and paragraph classification. (**b**) The top panel shows the schematic diagram of the standard text mining process: <i> expert annotation to build a baseline corpus; <ii> extraction of critical information from the literature text and construction of an extended corpus; <iii> store in a database for future data mining. The bottom panel shows an example of converting a synthesis sentence into action sequences. The key components of an action sequence such as starting and target material, synthesis steps and their conditions are found and extracted from the paragraph by different text mining algorithms (see Methods). (**c**) The entity types and their relationships extracted from the literature. The final constructed dataset can provide guidance for practical experimental work.

In recent work, our team has developed a text-mining pipeline to construct a dataset describing the CO_2_ reduction process catalyzed by copper-based electrocatalysts, which specifically includes material, regulation method, product, Faradaic efficiency and relevant conditions^12^. In the current work, we built a more advanced extraction pipeline based on the knowledge system of CO_2_ electrocatalytic reduction (Fig. 1), which uses various advanced machine learning, natural language processing techniques and large language models (LLMs) approaches to extract relevant information about the CO_2_ electrocatalytic reduction process from scientific literature. In addition, for the purpose of providing a more detailed and complete guidance scheme for materials scientists to develop new catalysts, we designed a set of synthesis actions with predefined properties and a deep-learning sequence to sequence model based on the transformer architecture, which converts unstructured experimental procedure text into structured action sequences. By applying the extraction pipeline, we present an open-source corpus for electrocatalytic CO_2_ reduction. The database contains two types of corpus: (1) the benchmark corpus, which is a collection of 6,086 records extracted from 1,081 publications by catalysis postgraduates; and (2) the extended corpus, which consists of content extracted from the abstract of 5,941 documents using traditional natural language processing techniques and large language models techniques. Respectively, the Extended Corpus I contains 77,016 records and the Extended Corpus II contains 30,283 records. In addition, we extracted 476 synthesis procedures for catalytic materials from 2,176 full-text documents, and the extracted information includes target and preparation materials, synthesis operations and the quantity of materials involved in them, and operation properties. The Extended Corpus was evaluated and revised by domain experts. This work provides a valuable resource to accelerate research into CO_2_ reduction by supplying structured information and datasets ready for further analysis and hypothesis generation. The tools and datasets created could significantly reduce the time and resources required for literature review and data gathering, allowing scientists to focus on innovation and experimentation.

## Methods

The schematic overview of the extraction pipeline is shown in Fig. 1. We first searched the literature related to the electrocatalytic CO_2_ reduction process following a series of filtering criteria. For scientific article retrieval and preprocessing, the raw archived corpus was parsed and organized in paragraphs. After paragraph classification, the paragraphs related to the concrete synthesis procedures were automatically selected. The extracted information includes the materials, the target products, their quantities as well as the synthesis operations and their attributes. We then constructed action sequences for each synthesis action in a predefined format. Finally based on the the system of knowledge defined by domain experts, we published a manually annotated baseline corpus and an automatically annotated extended corpus. The final generated dataset can be used for domain data mining and further downstream NLP tasks, as well as provide guidance to material domain scientists for practical experimental work.

### Content acquisition

Scientific publications used in this work are journal articles published by Elsevier, the Royal Society of Chemistry, American Chemical Society, Wiley, Acta Physico-Chimica Sinica & University Chemistry Editorial Office (Peking University), MDPI, the Electrochemical Society, Springer Nature, etc. For each publisher, the journals relevant to materials science were manually selected. We used regular expression matching^20^ to obtain the dois of relevant literature in the field of CO_2_ electrocatalytic reduction. Specifically, we searched and exported metadata for more than 27,000 articles by using the keywords “CO_2_”, “Reduction”, and “Electro*” as subject indexes on the Web of Science website. The exported literature metadata was then filtered step by step according to expert-defined rules. The title of every article was queried for words “CO_2_”, “carbon dioxide” or “CO(2)”, which yielded 9,850 articles. The abstract of every article was queried for words “electroc” or “electror”, which yielded 6,973 articles. Finally the domain experts performed manual filtration to exclude articles whose titles contained words that were not relevant to the topic, including: “photoc”, “light”, “visible”, “solar”, “microbial”, “bacteria”, “culture”, etc. we eventually obtained 5,941 summary texts of the literature related to the work on CO_2_ electrocatalytic reduction and scraped the full text of 2,776 papers from the web. We finally acquired the literature in PDF format and used the PyMuPDF tool, a PDF parsing tool^21^, to automatically process these literature data to obtain their metadata such as title, authors, abstract, etc. and the full text in json format. Since the processed document contains irrelevant tags, we developed a data cleaning method for parsing the article tag strings into consistently formatted text paragraphs while retaining the same chapter and paragraph structure as the original paper.

### Paragraph classification

We used the Transformers Bidirectional Encoder Representation (BERT) model to identify paragraphs containing descriptions of synthesis methods. MatBERT is a BERT model^22^ specifically for material science texts, pre-trained on over 2 million papers in a self-supervised manner, i.e. by predicting masked words based on the context around the target sentence. After training the BERT model, we used a paragraph classification method based on semi-supervised learning^23^. First we applied latent Dirichlet allocation (LDA)^24^ on the 12,643 articles in the field of photoelectrocatalysis to identify the experimental steps implicit in sentences. Then we collected all the paragraphs from the literature and manually labelled the paragraphs describing the synthesis protocol. The training data ultimately included 760 training examples, with 228 positive examples and 532 negative examples. We applied the random decision forest (RF) algorithm^25^, a supervised machine learning method, to binary classify the training data. This step yielded 476 synthesis paragraphs from a total of 2,776 articles.

### Entity annotation

In order to improve the quality of the training data based on the automatically extracted models, we generated a higher-quality dataset, also known as a *gold standard corpus*^26^, by manually annotating a portion of the sentences from the abstracts and body of literature related to CO_2_ electroreduction. We developed an annotation framework based on the doccano annotation tool^27^. Annotators can open the framework in a web browser and browse through the sentences of the material literature. The page displays the sentence to be annotated along with predefined entity types and related descriptions. The annotator can add new entities, reorder them or edit them by opening a separate view. To ensure consistency between annotators, detailed annotation guidelines are provided.

### Entity extraction

In our previous study, we extracted nine types of entities in the literature based on the constructed electrocatalytic reduction system, including material, regulation method, product, faradaic efficiency, cell setup, electrolyte, synthesis method, current density, and voltage. Some of these entity labels are provided with more detailed labelling subclasses to ensure that materials scientists have access to more complete information. In the current construction of the CO_2_ electrocatalysis literature dataset, We have updated the categories of the tag subcategories according to the new knowledge system. In addition, we added information on the material synthesis process, which converted unstructured scientific paragraphs describing catalytic materials synthesis into pre-defined “coded recipes” of synthesis. The recipes includes not only the starting materials and final target products but also the synthesis actions and their attributes.

### Construction of extended corpus

Traditional entity extraction methods follow the pattern of “expert annotation, model training, model application” and use automatic extraction models to build a wider and larger corpus of lower quality, also known as a *silver standard corpus*(SSC)^26^. The Large Language Models (LLMs) such as GPT-3, GPT-3.5, and GPT-4 have been used for this purpose^28–30^. Its emergency provides a new paradigm for natural language processing modelling, i.e., building prompts with a small amount of expert annotation to directly fine-tune GPT models that have been pre-trained on large-scale data. Traditional NER methods are less general, but have higher domain confidence, while large models may produce uncontrollable illusions. Herein, in this paper, we used two model training approaches separately to generate an extended corpus based on the construction standard of the *silver standard corpus*(SSC).

#### Entity extraction using traditional NER methods

Regarding the hierarchical structure of entity labelling, we designed a two-step entity recognition model which consists of coarse-grained entity recognition and fine-grained entity classification. In the first step, we used the SciBERT model^31^ to convert each word token into an embedding vector. The embedding vector was then passed to a bi-directional long-short-term memory neural network with a conditional random-field top layer(BiLSTM-CRF)^32,33^ to identify which class of entity labels the corresponding token was. Considering that the representations of some entities usually have regularities, such as the chemical formula expressions of material entities and the numerical expressions of faradaic efficiency entities, we proposed a regular rule-based approach to assist the deep learning model^34^. The results of the two models were selected using a voting scheme^26^. In the second step, each coarse-grained type entity was classified into finer-grained entity classes using a classification algorithm combining dictionary and maximum entropy model. The dictionary-based recognizers used lists of words built on expert-annotated data^35^. For data that cannot be matched, the word embedding vectors, context vectors, word cluster clustering information and coarse-grained entity category information for each entity were passed through a simple mapping function. The final mapping results were used as entity features for classification probability prediction through a maximum entropy model.

**Fig. 2:**
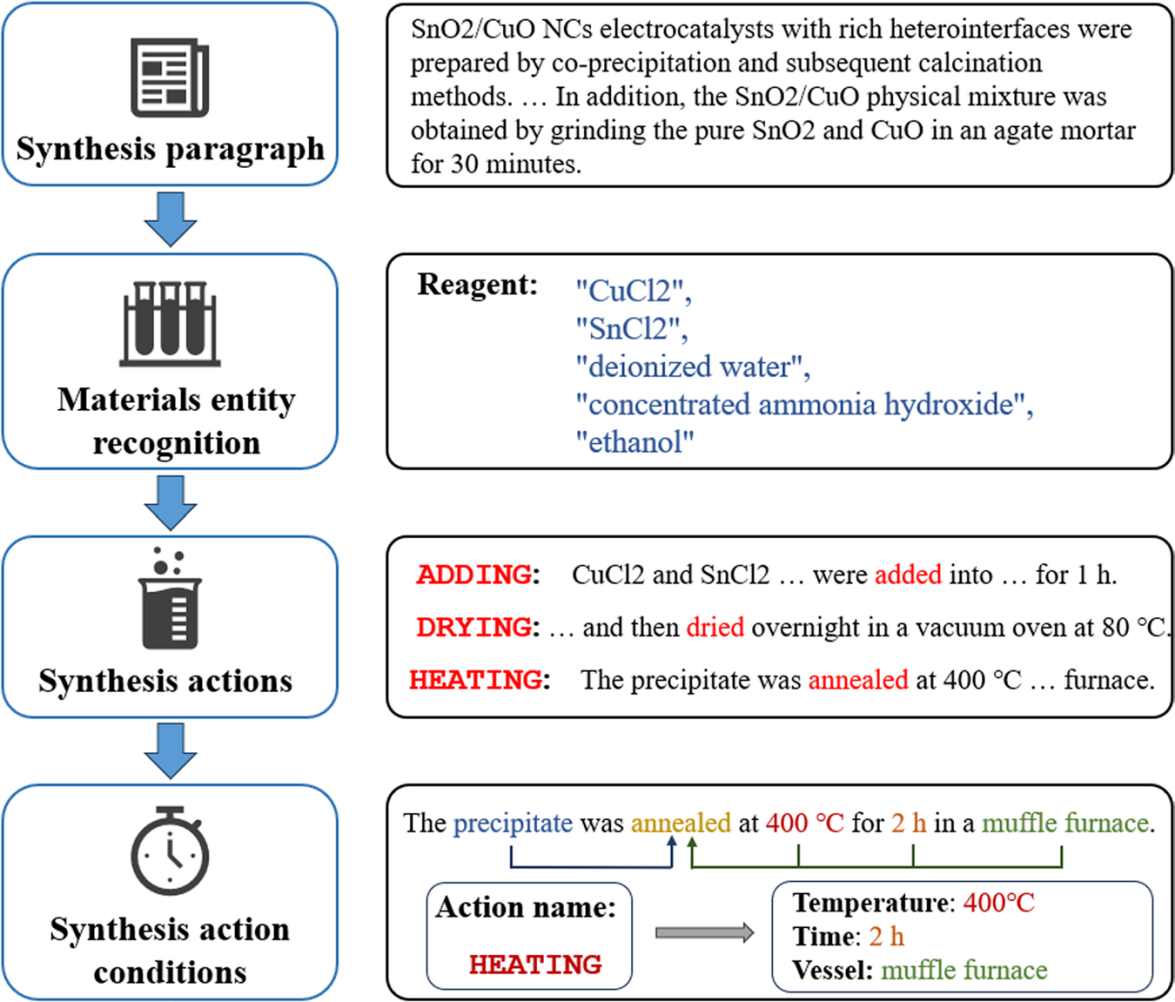
Schematic diagram of the process of converting a synthetic paragraph into action sequences.

A typical synthesis procedure in the electrocatalytic reduction literature contains information on the prepared and target materials, synthesis operations and operating conditions. These items are organized into material synthesis “recipes” and are extracted from the synthesis paragraph as shown in Fig. 2. Our extraction process consists of multiple algorithms that analyze the passages and identify the relevant materials, the synthesis actions performed, and the condition information associated with those synthetic actions. The method used in each step of the extraction process is described in detail below.

**Step 1: Materials entity recognition**. The first step is the labelling of the preparation material. The synthesis of the target material involves the names of all the reagents that need to be prepared. We used pattern matching against a database of common reagent names and then used a plain Bayesian classifier to determine whether a candidate phrase is a reagent name, excluding some specific phrases^36^. Through iterative trials, we eventually chose reagent names from the Reaxys database and non-reagent-name texts from the Brown English language corpus to train the classifier.

**Step 2: Synthesis actions** To identify and classify synthesis actions described in passages, we implemented an algorithm that combines Recurrent Neural Networks (RNN) and rule-based sentence dependency tree parsing^22^. The neural network labelled the sentences in the synthetic passages into nine categories: NOT OPERATION, ADDING, HEATING, CURING, ELECTROCHEMICAL ANODIZATION, FILTERING, DRYING, DIPPING and REACITON, which are the main operations in catalytic materials synthesis. We used ChemDataExtractor’s ChemWordTokenizer^37^ to tokenize the lemmatized sentences. For each synthesis action obtained, we used the SpaCy library^38^ to parse the syntactic information of the dependency subtree for linguistic features of the tokens, such as their lexical properties and their dependency on the root token.

**Step 3: Synthesis action conditions** For each synthesis action, we used dependency tree parsing and rule-based regular expression methods^39^ to extract the relevant attributes of the synthesis action, such as heating time, heating temperature, and potential voltage values. In addition, if there were materials involved such as ADDING and REACTION operations, we used pattern-matching techniques to extract the names and corresponding quantities of the reagents involved. For example, one of the patterns used for finding solutions is “a/an XX solution containing Reagent” in which “Reagent” represents a phrase previously tagged as a reagent. An example phrase that would be matched by this pattern is “an aqueous solution containing HAuCl_4_(10 mol, 125 mL)”. The contents of the parentheses are regularly matched to the corresponding quantities of the reagents.

#### Entity extraction using LLMs

In previous study, we attempted to construct a corpus using an NLP model, but the accuracy of the intelligent model is easily affected by the volume of training data. Herein, we demonstrate that LLMs, including original LLMs and fine-tuned LLMs, can act as assistants to collaborate with human researchers, facilitating entity recognition and text mining to accelerate the research process.

In the realm of catalyst-related tasks, LLM’s performance can be significantly enhanced by employing prompt engineering (PE) which can steer LLMs toward generating precise and pertinent information. Although LLMs, including fine-tuned LLMs, can answer general questions, their knowledge depth, accuracy and timeliness are limited in vertical domain filed. To solve this problem, we use vector databases to enhance the reasoning ability of LLMs in vertical domains. Vector databases can transform literature and data into vector representations by embedding vectors. Sci-BERT^31^ was used as embedding model for construct the vector database.

**Fig. 3:**
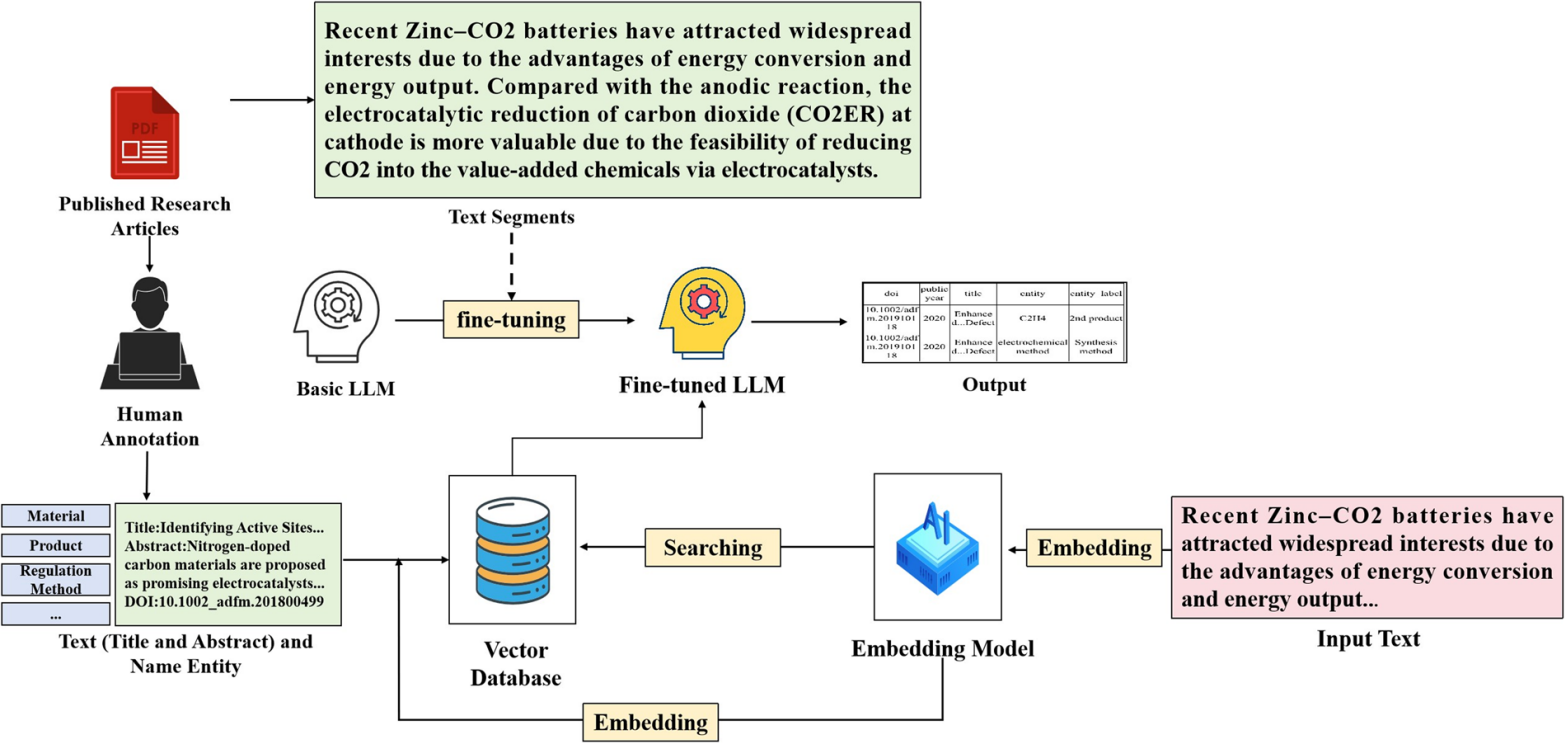
The schematic overview of extraction using LLMs and vector database.

**Fig. 4:**
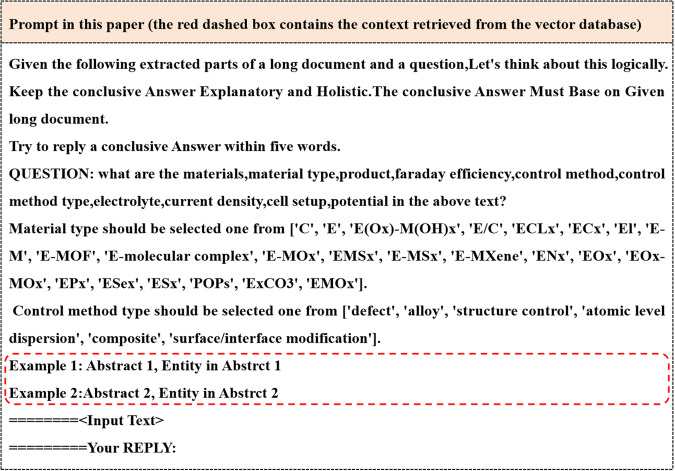
The prompt using in the entity extraction.

Figure 3 shows the process of knowledge extraction using LLMs and vector database. Firstly, we processed and cleaned the full text of 12,643 photoelectrocatalytic scientific literature, and used them for LLMs fine-tuning. In this step, we chose Vicuna-33b-v1.3 as the basic LLMs. Secondly, we extracted the title, abstract and doi from articles associated with standard corpus, then we use Sci-BERT as the embedding model to transform title and abstract into vector. When performing entity recognition, user first input the text to be extracted, embedding model transform it into vectors. Then the similar articles will be obtained by calculating the vector distance, and will be used to generate precise and pertinent information, which be shown in Fig. 4. The prompt will be input to the fine-tuned LLMs for entity recognition.

## Data Records

The both types of datasets constructed in this work are available in ScienceDB, a public, general-purpose data repository designed to serve data to researchers, research projects/teams, journals, institutions, universities, and others. The metadata contained in the article dataset includes: article DOI, year of publication, and title. Each record corresponds to the process of CO_2_ electrocatalytic reduction and its metadata includes: the entity extracted from the paper, the label of the entity, and the sentence in which the entity is located. In addition, the datasets for the catalytic material synthesis methods are available as a single json. Each record corresponds to a synthesis procedure extracted from a paragraph and is represented as a separate json object. The metadata for each reaction includes the DOI of the paper from which the reaction is extracted as well as a fragment of the corresponding synthesis paragraph, the target product, the preparative material used in the reaction, and a tree of seven types of synthesis operations and their corresponding conditions. Table 1 gives extended details of all the dataset format.

**Table 1:** Format of each data record: description, key label, data type.

Data Description	Data Key Label	Data Type
DOI of the original paper	doi	string
Title of the original paper	title	string
Entity extracted from the paper	entity	string
Label of the entity	entity_label	string
Sentence where the entity is located	context	string
Target material data	target_string	list of strings
Equipment where the reaction is operated	hardware	list of strings
Material data of the preparation process	reagent	list of strings
Sequence of synthesis steps and corresponding conditions	operation	list of Objects (dict):
		-string: string
		-vessel: string
		-reagent: list of Objects^*a*^
		-speed: string
		-temp: list of Objects^*b*^
		-time: list of Objects^*b*^
		-potential: list of Objects^*b*^
		-condition: string
		-stir: boolean
		-reflux: boolean

The sequence of synthesis steps for the reaction (if specified in a paragraph) is listed as a data structure with the following fields: the original paragraph in the text (synthesis_paragraph), its type (operation_string) specified by the classification algorithm (see Methods), and the conditions associated with this operation step (conditions). We classified the types of operations involved in the synthesis of catalyst materials into eight categories and give detailed descriptions of the types of operations and condition attributes in Table 2.

**Table 2:** Format of each synthesis operation record: operation type, condition attributes, data description.

Operation Type	Condition attributes	Data description
**ADDING**	-left_reagent	The name and quantity of materials involved in the operation
	-right_reagent	The name and quantity of materials involved in the operation
	-speed	Speed of adding operations
	-stir	Stirring or not during addition operation
**HEATING**	-vessel	Vessel in which the heating operation takes place
	-temp	Final temperature for heating operations
	-time	Time for heating operations
	-stir	Stirring or not during heating
	-reflux	Whether the heating process requires reflux
**CURING**	-condition	Curing conditions, deliberately stated in the paragraph
	-temp	Temperature during curing operation
	-time	Time for curing
**ELECTROCHEMICAL ANODIZATION**	-reagent	Name of electrode solution
	-potential	Potential values for anodic oxidation reactions
	-time	Time for the electrochemical anodization
**FILTERING**	-condition	Filter conditions, the original sentence text extracted directly
	-reagent	Name of the reagent being filtered
**DRYING**	-condition	Dry conditions, the original sentence text extracted directly
**DIPPING**	-left_reagent	Name of material to be dipped
	-right_reagent	Name of material immersed in
	-time	Time for the immersion
**REACTION**	-left_reagent	Name and quantity of materials involved in the reaction
	-right_reagent	Name and quantity of materials involved in the reaction
	-temp	Temperature at the time of reaction
	-time	Time for the reaction
	-reflux	Whether reflux is required for the reaction

The corpus is publicly available at Science Data Bank (ScienceDB), which is a public, general-purpose data repository aiming to provide data services for researchers, research projects/teams, journals, institutions, universities, etc. The benchmark corpus is publicly available at 10.57760/sciencedb.13290^40^. The extended corpus I and extended corpus II are publicly available at 10.57760/sciencedb.13292^41^, where include other extendedcorpuscorpus exacted by LLM model. The two types of Corpus are provided as a file in CSV format, and the details of them are shown in Table 3. A complete dataset of 476 catalytic material synthesis processes is publicly available at 10.57760/sciencedb.13293^42^.

**Table 3:** Summary of the three corpus.

Corpus Type	Benchmark Corpus	Extended Corpus I	Extended Corpus II
Entity Type			
Material	1,092	18,184	5,977
Regulation method	1,086	35,780	5,488
Product (including the second and third product)	1,340	19,080	6,700
Faradaic efficiency (including the Faradaic efficiency of second and third product)	1,135	3,496	3,152
Cell setup	435	—	170
Electrolyte	475	—	3,919
Synthesis method	228	476	—
Current density	393	—	3,852
Voltage	801	—	1,025
Total	6,985	77,016	30,283

## Technical Validation

### Extraction accuracy

To demonstrate the utility of the extended corpus, we first evaluated the model against other current state-of-the-art traditional entity extraction methods. We selected several generic neural network tagging models, including bi-directional LSTM layers with conditional random field (CRF) layer^33,43,44^, bi-directional recurrent neural network Bi-GRU^45^, and BERT model with CRF layer. We then chose a multi-feature based maximum entropy machine learning model^46^ using two types of features, Parts-of-Speech features generated by GENIA Parts-of-Speech Tagger^47^and lexical features. Table 4 shows the results of the experimental comparison. We found that our constructed entity extraction model consistently outperforms other methods, achieving an overall F1 score of 85.16 in recognizing four coarse-grained categories of entities. This also demonstrated an advantage in the subsequent classification of fine-grained entities.

**Table 4:** Compare the F1 scores of entity recognition in various models.

Entity(freq. in test set)	MaxEnt	BiLSTM-CRF	BiGRU-CRF	BERT-CRF	BERT-BiLSTM-CRF
MATERIAL(92)	40.12	50.43	52.01	59.96	**60**.**59**
METHOD(97)	38.25	46.89	49.67	57.12	**58**.**02**
PRODUCT(94)	70.21	82.45	86.12	91.10	**92**.**34**
FARADAIC EFFICIENCY(62)	88.16	91.18	91.98	94.56	**96**.**82**
Macro-avg F1	51.26	66.90	68.02	71.48	**73**.**90**
Micro-avg F1	68.89	81.02	82.33	82.73	**85**.**16**

To estimate the quality of the synthesis process dataset, we had a human expert test 100 randomly selected entries. The human expert manually extracted the information provided in the synthesis paragraphs and compared the results with those extracted by the pipeline. Table 5 presents the accuracy statistics, which include the precision, recall, and F1 scores calculated from the test entries.

**Table 5:** Accuracy of synthesis information extraction models.

Pipeline Component	Extraction Method	F1:(precision | recall)
Article filtering	Regular match	0.88:(0.84 | 0.93)
Synthesis paragraph classification	BERT classification	0.80:(0.82 | 0.78)
Materials entity recognition	BiLSTM + CRF (BERT embedding) & Regular match	0.96:(0.98 | 0.94) - materials
		0.84:(0.86 | 0.82) - targets
Synthesis actions	BiLSTM (Word2Vec embeddings)	0.89: (0.92 | 0.86)
Synthesis conditions	Rule-based	
-Temperature		0.95: (0.98 | 0.93)
-Time		0.96: (0.98 | 0.94)
-Potential		0.88: (0.91 | 0.86)
Material quantities	Rule-based	0.90: (0.93 | 0.87)

We also validated the entity recognition results of the LLMs in this paper. We validate the answers of the LLMs by an expert with 160 randomly selected entries, and ensure that each category has 20 test data. The evaluation result is shown in Table 6. The *Count* means the total amount of samples from different categories, the *Correct* means the number of correctly identified entities, and the *Existence* means the number of entities of this type does exist in the text input to the large model. It is worth mentioning that if there is indeed no corresponding entity in the text input to the large model, the situation where the large model answers empty should also be considered as correct recognition. Therefore, we use *Modified Correct* to remove the above influence. Ultimately, we utilize *Modified Correct* and *Count* to calculate the evaluation of LLMs, which is *Modified accuracy*. Using large models for entity recognition also causes significant time loss. We used two NVIDIA A100 GPU graphics processing units for entity recognition, and cost almost 10 hours to process 5,941 literature abstracts.

**Table 6:** The evaluation of entity recognition of LLMs.

Entity	Count	Correct	Existence	Modified Correct	Modified accuracy
MATERIAL	20	15	17	15	75%
METHOD	20	13	19	13	65%
PRODUCT	20	17	17	17	85%
FARADAIC EFFICIENCY	20	11	11	18	90%
ELECTROLYTE	20	9	10	10	50%
POTENTIAL	20	7	7	16	80%
CURRENT DENSITY	20	7	7	12	60%
CELL SETUP	20	6	6	9	45%
OVERALL	160	85	94	110	68.75%

From the results, we can see that the LLMs perform better in entity extraction for numerical classes (faradaic efficiency, potential, etc.), but perform poorly in entity extraction for descriptive classes. This may be due to the objectivity of data entities, which reduces the possibility of hallucinations in large models.

### Dataset mining

**Fig. 5:**
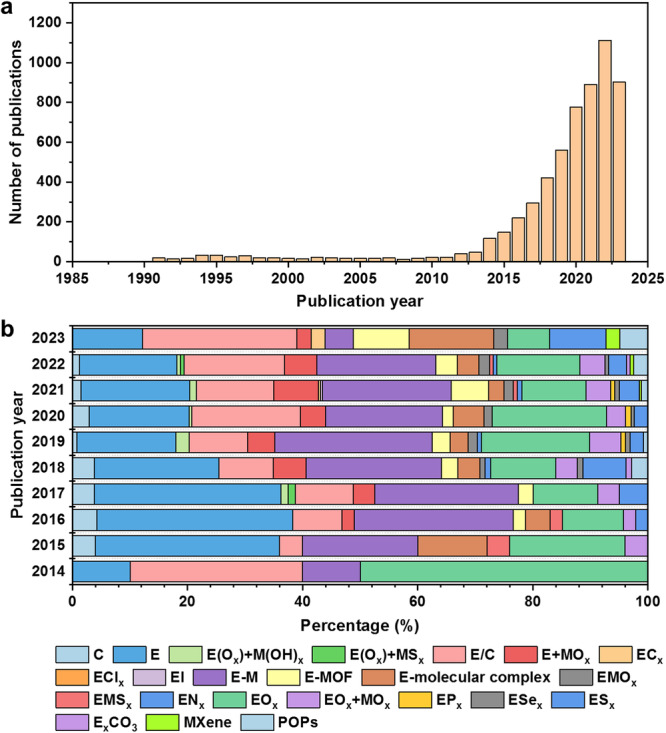
(**a**) Histograms of the number of publications of CO_2_ reduction electrocatalysts over the past thirty years. (**b**) Stacked histograms of the percentage of CO_2_ reduction electrocatalysts in the last ten years.

To present the recent trends in the development of CO_2_ reduction electrocatalysts, we showcased and analyzed the information in the database. Firstly, we demonstrated the publication trends of CO_2_ reduction electrocatalysts over the past 30 years. As depicted in Fig. 5a, articles on CO_2_ reduction electrocatalysts have experienced a rapid surge since 2010, indicating the burgeoning interest of scientists in this field. Figure 5b illustrates the proportional distribution of various types of CO_2_ reduction electrocatalysts. It is evident that the current research predominantly focuses on E (single metal), E/C (metal-carbon composites), E-M (binary or ternary metal systems), and EO_*x*_ (metal oxides), with a notable increase in attention toward E/C in recent years.

**Fig. 6:**
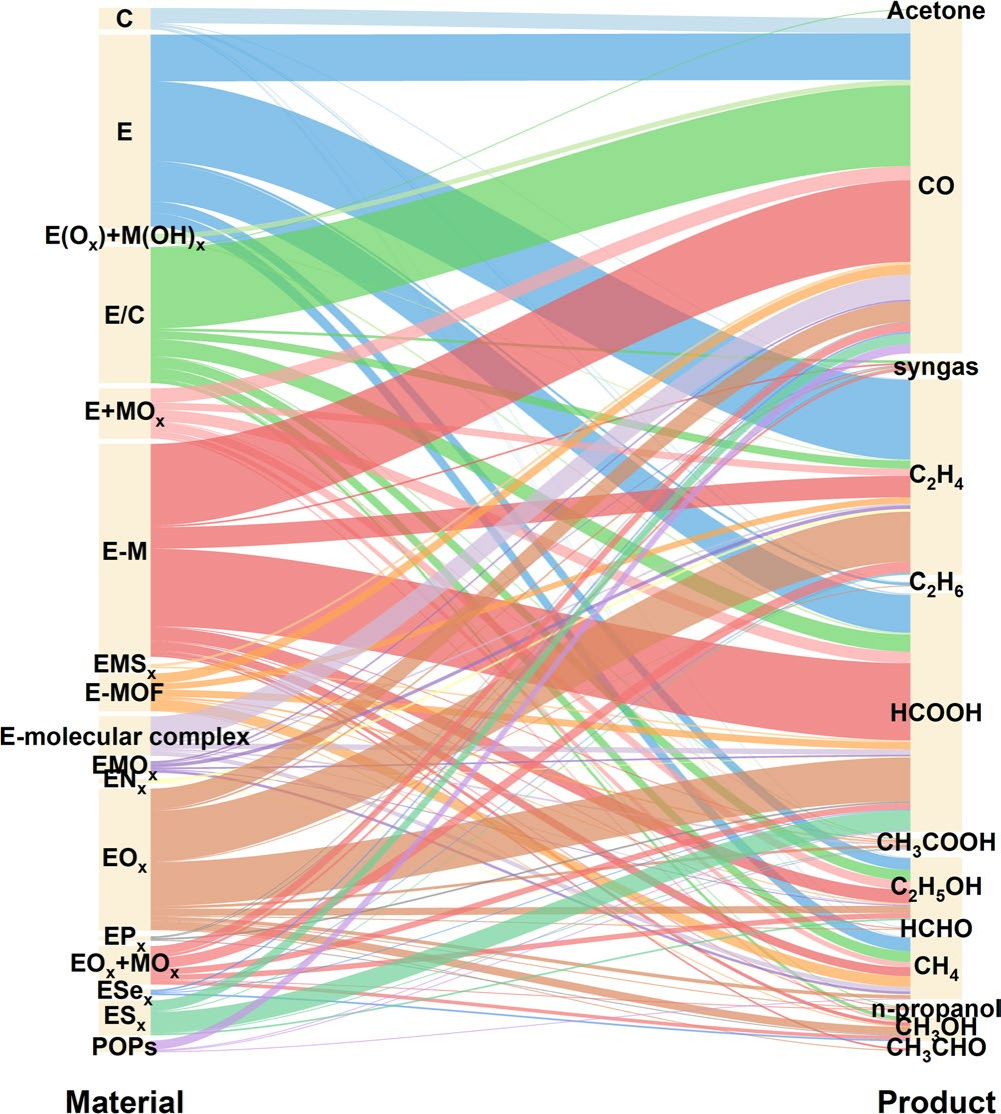
Alluvial plot illustrating the relationships between catalysts and products.

In addition to the overall development of electrocatalysts, another intriguing aspect lies in the correlation between catalysts and products, which is crucial for product-oriented catalyst design. Figure 6 presents an alluvial plot illustrating the intricate associations between catalysts and products. Notably, for clarity, less reported catalyst categories have not been included. E/C and E-M are favorable choices for generating CO, while E-M and EO_*x*_ exhibit the capability for formic acid production. For C_2_ products, such as C_2_H_4_ and C_2_H_5_OH, both E and EO_*x*_ are viable options. Furthermore, Fig. 6 also reveals some potential research topics that warrant further exploration. For instance, although a few catalysts demonstrate the ability to produce C_3_ products, such as n-propanol and acetone, the optimal catalysts have yet to be well-established. While composite systems are gaining increasing attention, their advantages over individual compounds remain to be fully elucidated.

**Fig. 7:**
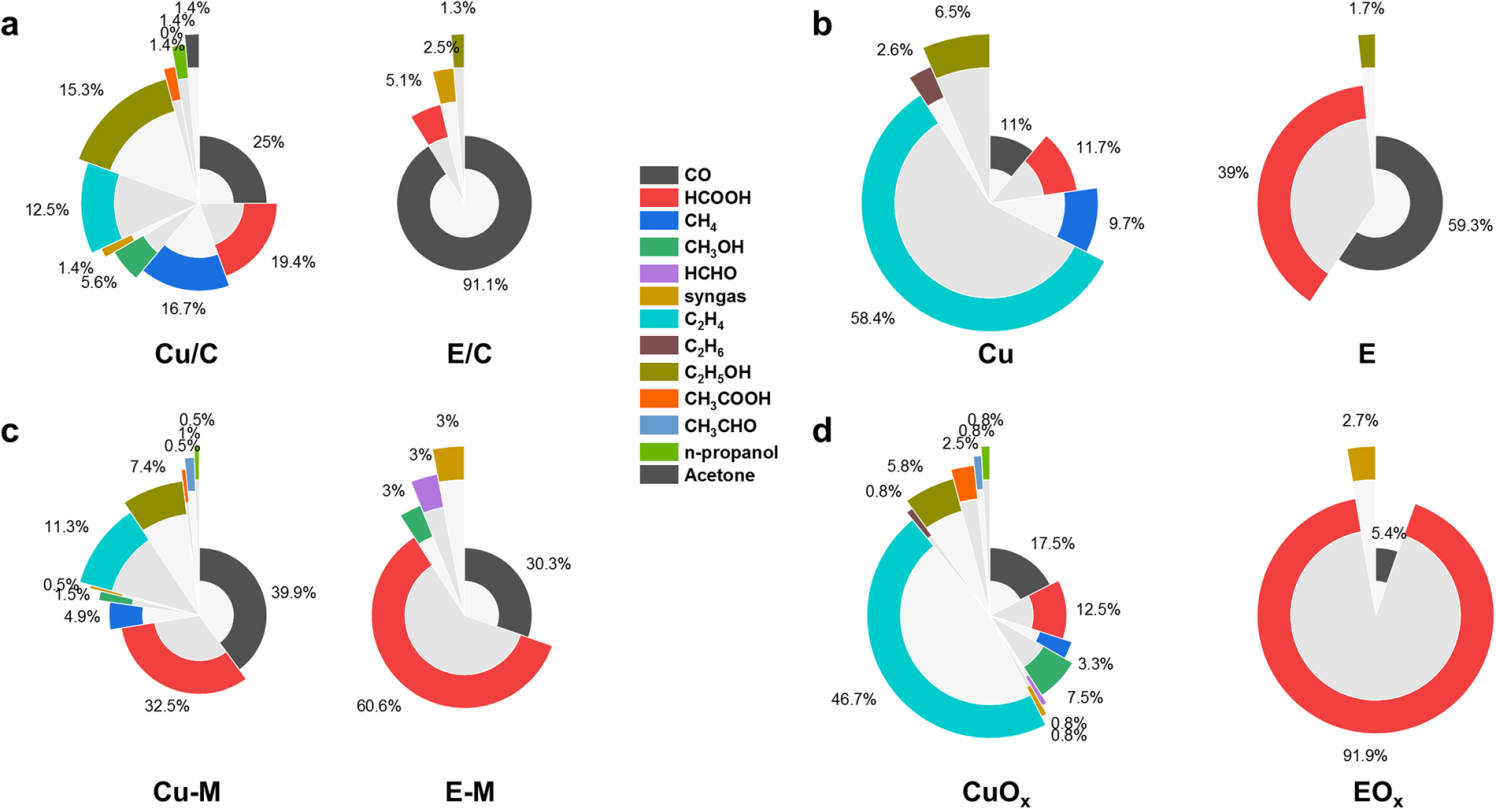
Doughnut charts showing the percentage of different products of catalysts with or without Cu.

Moreover, the type of metal, particularly the presence of Cu, is crucial for the performance of catalysts in CO_2_ electroreduction. Therefore, we annotated whether the catalysts contained Cu in the database. To illustrate this contrast clearly, we generated doughnut charts to display the percentage of different products from several types of catalysts with or without Cu. As shown in Fig. 7a, the majority of the products for E/C are CO, while Cu/C can generate various C_1_ and C_2_ products. For single metal systems (Fig. 7b), the primary products of E are C_1_ products, whereas Cu yields predominantly C_2_ products. In the case of binary or ternary metal systems, Cu-M exhibits a stronger capability for producing C_2_ products compared to E-M. Regarding metal oxides, the products of EO_*x*_ are predominantly formic acid, while CuO_*x*_ yields primarily C_2_H_4_. These findings underscore the significant impact of the presence of Cu on the selectivity of C_2_ products for catalysts.

**Fig. 8:**
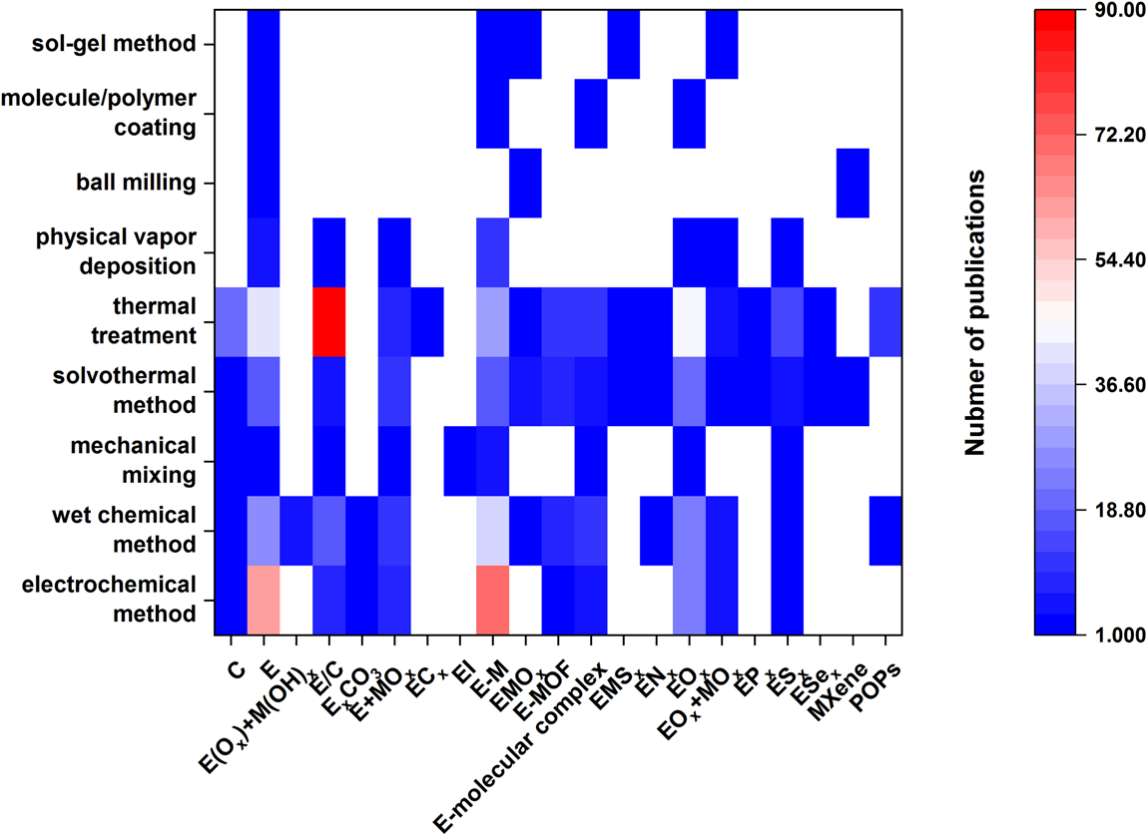
Heatmap showing the number of publications of CO_2_ electrocatalysts with different synthesis methods.

The choice of synthesis method also has a significant impact on the performance of catalysts, so we analyzed the correlation between catalysts and synthesis methods. As shown in Fig. 8, thermal treatment and solvothermal methods are the two most widely used material synthesis methods. In addition, different catalysts also have their conventional synthesis methods. For example, the synthesis of Cu/C, which usually refers to carbon-coated metal nanoparticles or anchored single atoms, is mainly through thermal treatment. The synthesis of E and E-M is mainly electrochemical methods, especially electrochemical reduction treatment. For EO_*x*_ and its composites, the solvothermal method, wet chemical method, and electrochemical method are commonly used methods. This analysis is helpful for the screening of target catalyst synthesis methods.

The database encompasses various catalyst types and diverse regulation strategies, which can be utilized to guide the design and optimization of novel catalysts. One feasible approach involves integrating multiple strategies by drawing inspiration from well-performing catalysts and regulation methods in the literature, thus facilitating the development of highly efficient catalysts. For example, CuS serves as a potential efficient catalyst for C_2_H_4_ production, while nano-sized polymer coatings can enhance the selectivity of C_2_H_4_. Consequently, CuS nanoparticles coated with an a-few-nm-thick polymer layer represent an effective method for selectively producing C_2_H_4_. Similarly, coupling Cu_2_O nanocrystals with (111) facets with functionalized graphene nanosheets can be employed for C_2_H_5_OH production. Furthermore, utilizing fine-tuned domain LLMs is also a viable strategy for developing novel catalysts, and further efforts are required in fine-tuning LLMs and prompt engineering.

## Data Availability

The scripts utilized to parse articles and extract entities are home-written codes which are publicly available at the github repository https://github.com/cxqwindy/CO2_reduction_electrocatalysts_db. The underlying machine-learning libraries used in this project are all open-source: rxn4chemistry(rxn4chemistry), ChemDataExtractor (chemdataextractor.org)^37^, gensim (radimrehurek.com)^48^, PyMuPDF(PyMuPDF), Pytorch (www.pytorch.org) and scikit-learn (scikit-learn.org)^49^.
